# Gout in an Obese Patient with Nonalcoholic Steatohepatitis on a Thiazide Diuretic and Association Between Hyperuricemia and Nonalcoholic Steatohepatitis: A Case Report

**DOI:** 10.7759/cureus.39207

**Published:** 2023-05-18

**Authors:** Zahid Khan, Amresh Gul

**Affiliations:** 1 Acute Medicine, Mid and South Essex National Health Service (NHS) Foundation Trust, Southend-on-Sea, GBR; 2 Cardiology, Barts Heart Centre, London, GBR; 3 Cardiology and General Medicine, Barking, Havering and Redbridge University Hospitals National Health Service (NHS) Trust, London, GBR; 4 Cardiology, Royal Free Hospital, London, GBR; 5 General Practice, Lifeline Hospital, Salalah, OMN

**Keywords:** thiazide diuretics induced gout, medication-induced gout, septic arthritis, thiazide diuretics, diabetes type 2, internal medicine and rheumatology, elevated uric acid levels, gout flare, gout crystals, gout disease

## Abstract

Gout is a common inflammatory arthritis caused by increased uric acid crystals in and around various joints, mainly the big toe in adults. It happens due to the increase of urate or uric acid levels either because of increased production or decreased excretion from the body. Uric acid is the final product of purine metabolism, and many patients with hyperuricemia may remain asymptomatic. We present a case of a 46-year-old male who presented to the ambulatory care unit with the clinical features of acute pharyngitis and left toe pain for the past three days. On further questioning, he added that he had pain in the left lumber region and left side of the toe for the past few months. He also had a known case of type 2 diabetes mellitus, hypertension, and gastritis, for which he has been taking the thiazide diuretic, angiotensin-converting enzyme (ACE) inhibitors, metformin, sitagliptin, aspirin, and atorvastatin. Laboratory tests showed elevated uric acid along with raised inflammatory markers. As a result, he was referred to the specialist for arthrocentesis in order to confirm the diagnosis, and the thiazide diuretic was replaced with calcium channel blockers. He also suffered from nonalcoholic steatohepatitis (NASH) based on his ultrasound abdomen. On the follow-up visit, his symptoms had resolved, and his uric acid level had normalized.

## Introduction

Hyperuricemia is a biochemical condition, a precursor of gout, defined as an elevated uric acid level >6.8 mg/dL (404 µmol/L) [1]. Elevated level of uric acid is a good predictor of underlying co-morbidities including diabetes, obesity, and hypertension [1,2]. Drug-induced hyperuricemia can be due to increased uric acid level because of reabsorption and/or decrease in uric acid secretion resulting in gout and is an increasingly common presenting problem in clinical practice [3]. Hyperuricemia is also caused by high level of alcohol consumption and intake of purine-high foods along with medications such as thiazide diuretics, loop diuretics, aspirin, cytotoxic chemotherapy, anti-tuberculous therapy, and testosterone [3]. Diuretics are one of the most important causes of secondary hyperuricemia. The use of loop diuretics, thiazide diuretics, and thiazide-like diuretics was associated with an increased risk of gout [4,5]. The exact incidence and prevalence of drug-induced hyperuricemia and gout are not clear, but one hospital study reported its prevalence to be about 20% [6]. Our 46-year-old patient was also taking thiazide diuretics for hypertension for a long time period and never had uric acid level checked on his routine clinical visits with his general practitioner (GP).

Hyperuricemia has been reported to be associated with nonalcoholic steatohepatitis (NASH) [7]. Several studies have demonstrated that baseline high serum uric acid levels were an independent predictor of the development of hepatosteatosis over time [8,9,10]. NASH has been reported to be closely associated with metabolic syndrome and its related conditions such as type 2 diabetes mellitus, hypertension, dyslipidemia, and hyperuricemia [11].

## Case presentation

**Figure 1:**
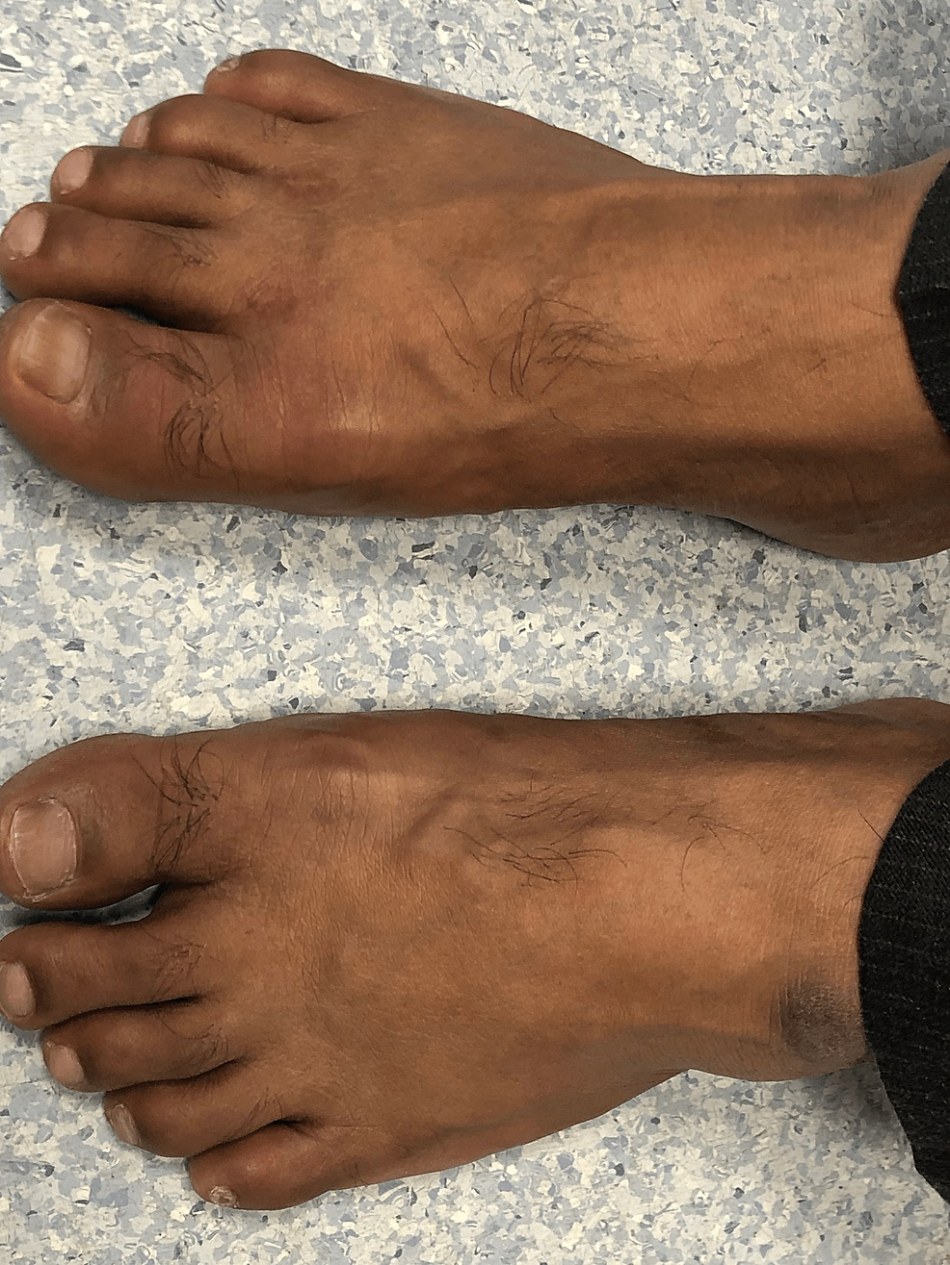
Swollen and red right big toe and normal left big toe

**Figure 2:**
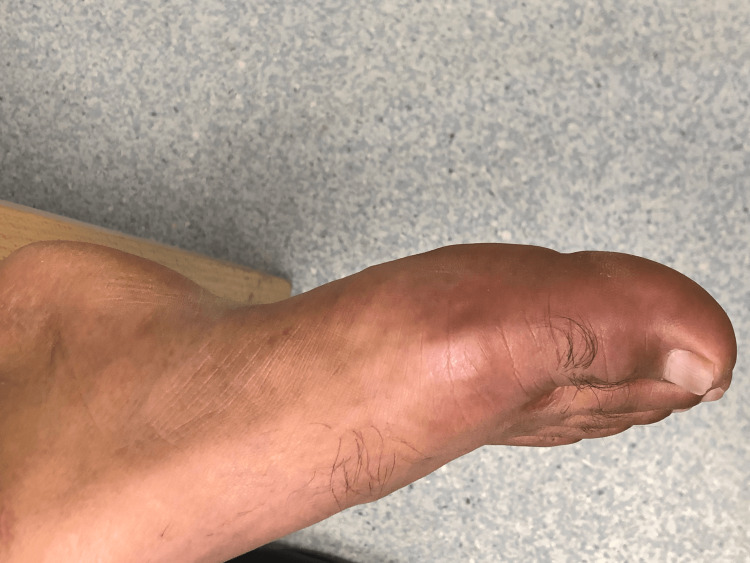
Swollen and red right big toe

**Figure 3:**
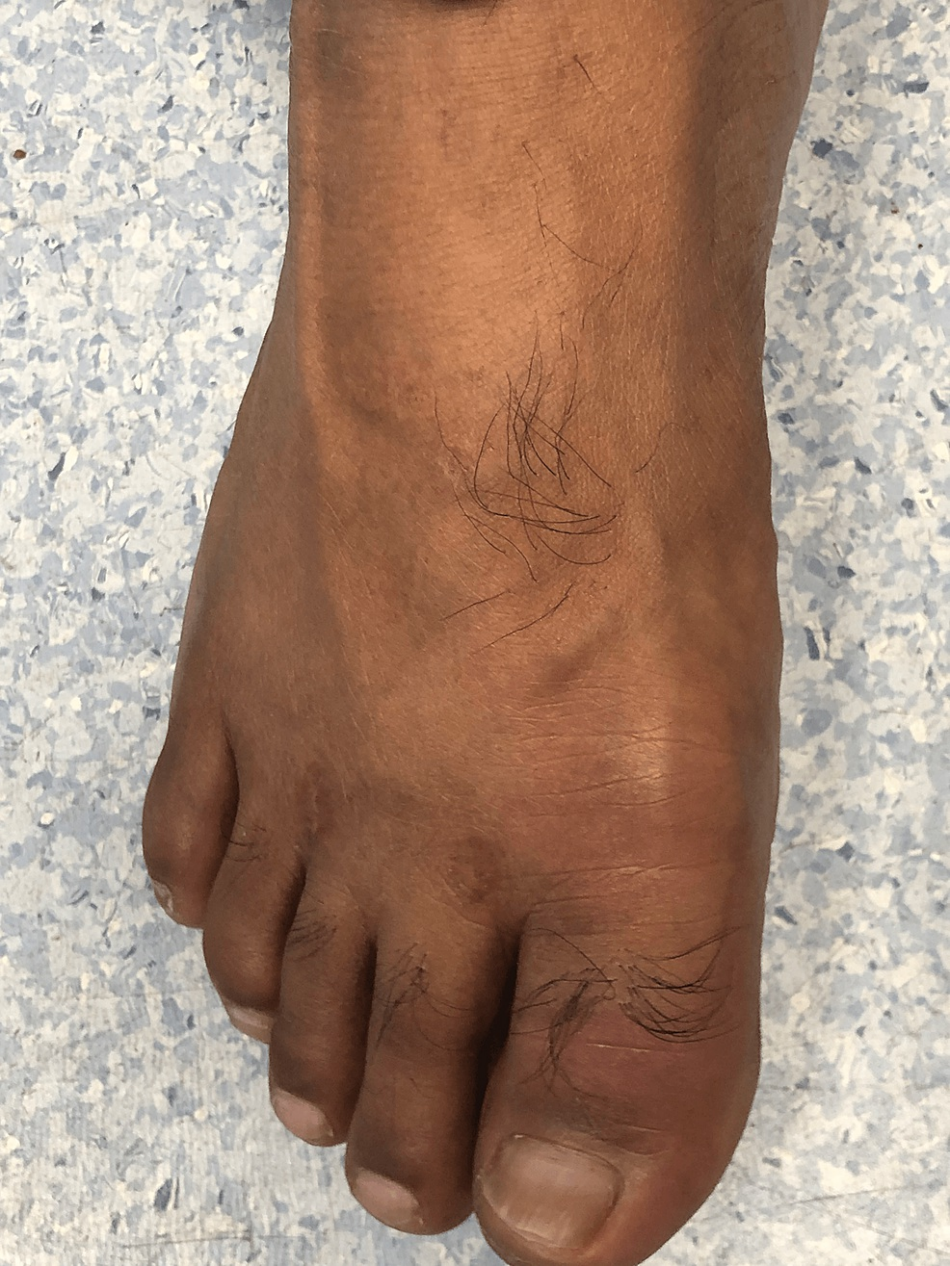
Inflamed right big toe

A 46-year-old male of middle eastern origin presented with complaints of sore throat, fever, productive cough for the last two days, and pain in the right big toe for the last three days. His past medical history was significant for high BMI, type 2 diabetes mellitus, hypertension, dyslipidemia, and gastritis. He also complained of on-and-off pain in the left lumber region and right big toe for the past few months that responded to over-the-counter ibuprofen without seeking any medical attention. His regular medications include thiazide diuretics, ACE inhibitors, metformin, sitagliptin, aspirin, and atorvastatin. Vitals signs showed a blood pressure of 120/78 mmHg, pulse of 87 bpm, and BMI of 30. There were swelling and tenderness of the right big toe with reduced flexion (Figures 1-3). His blood report showed elevated serum uric acid level of 8.7 mg/dL along with increased CRP and ESR (Table 1). He was referred to a rheumatologist and had arthrocentesis, which confirmed the presence of negatively birefringent needle-shaped monosodium urate (MSU) crystals, and thiazide diuretics was replaced by amlodipine 5 mg once a day. He was commenced on naproxen 500 mg twice daily and was also given a course of amoxicillin for acute pharyngitis. Following the resolution of his symptoms, he was commenced on allopurinol 100 mg once a day and was provided with dietary advice.

On subsequent visits, his symptoms of hyperuricemia were alleviated, and he felt much better with his new medication. He was taking aspirin for primary prevention, which was stopped due to gastritis symptoms and to minimize the risk of future gout flare-ups. He was also commenced on lansoprazole 30 mg once daily. He presented to the hospital after three months with a further flare-up of right big toe gout precipitated by increased consumption of red meat and fish and non-compliance with allopurinol after an initial improvement in his symptoms. He had inflamed, hot, and swollen right big toe again on admission. He was also having epigastric pain associated with nausea and vomiting, lethargy, and reduced appetite for the past few days. The blood report showed elevated uric acid of 9.7 mg/dL and elevated alanine transaminase level. Abdominal ultrasound confirmed the diagnosis of NASH, which has been reported to be associated with hyperuricemia. As a result, he was advised to do exercise to lose weight, do dietary modification, and exclude purine-rich foods, and also take medicine regularly. After one month of lifestyle modification, avoidance of purine-rich food, and strenuous exercise, he started feeling much better and his uric acid level came to normal along with improvement of his symptoms. 

## Discussion

Gout is a chronic disease that involves the deposition of MSU crystals in the body, in particular the joints, soft tissues, and kidneys. The main symptoms of gout are joint pain and swelling, which may present as an acute attack, chronic gouty arthritis, or acute attack on chronic gouty arthritis in a patient. The major manifestations of gout in the kidney are nephrolithiasis and chronic urate nephropathy, both of which can progress to chronic kidney disease [12,13].

Gout occurs when the serum uric acid concentration is sufficiently elevated (usually greater than 0.42 mmol/L [7 mg/dL]) leading to crystals formation in tissues. While the presence of hyperuricemia is important in the diagnosis of gout, patients with acute gout may have a normal serum uric acid concentration. Furthermore, the presence of hyperuricemia does not necessarily indicate that gout is the explanation for a patient's symptoms. Patients can also have asymptomatic hyperuricemia, which is a risk factor for developing gout. Gout can be effectively treated, and its complications can be prevented with adherence to lifelong urate-lowering therapy using a treat-to-target approach; however, gout is often poorly managed in Australian primary care, with low prescribing rates of allopurinol, poor uric acid monitoring, and low achievement of serum uric acid targets [14]. Patient adherence to urate-lowering therapy is also often suboptimal [14].

Uric acid is formed in the liver from dietary and endogenous purines. Consumption of purine-rich foods (particularly meat and seafood), alcohol (particularly beer and spirits), and fructose-sweetened drinks can increase serum uric acid concentration and the risk of gout in susceptible individuals. Disorders involving a high cell turnover, such as hematological malignancies and severe psoriasis, can also increase serum uric acid concentration and the risk of gout in susceptible individuals [15,16,17]. Uric acid is eliminated by the kidneys (two-thirds) and the gut (one-third). Drugs that inhibit the renal excretion of uric acid can increase serum uric acid concentration and the risk of gout in susceptible individuals. These drugs include thiazide diuretics (often taken as a combination product with an angiotensin-converting enzyme (ACE) inhibitor or angiotensin II receptor blocker for blood pressure control in the Western world), loop diuretics, and ciclosporin. Diuretics are the most important cause of secondary gout in middle-aged and older people [15,16]. Comorbidities including hypertension, chronic kidney disease, dyslipidemia, type 2 diabetes, and obesity are risk factors for hyperuricemia which in turn is associated with NASH. A high concentration of endogenous insulin, as seen in patients with obesity, also inhibits the renal excretion of uric acid. A five-year cohort study demonstrated that hyperuricemia was associated with a higher incidence of NASH. The baseline uric acid levels for patients were categorized into the following quartiles: 0.6-3.9 mg/dL, 3.9-4.8 mg/dL, 4.8-5.9 mg/dL, and 5.9-12.6 mg/dL, and the incidence of NASH with baseline uric acid increased to 5.6%, 9.8%, 16.2%, and 20.9%, respectively, after five years. This association was confirmed by multiple logistic regression analyses demonstrating that hyperuricemia was associated with the development of NASH [18]. Xu et al. (2020) with 813 participants (605 males and 208 females) demonstrated that baseline serum uric acid level predicted the incidence of NASH in both men and women over 3 years period [10].

Essentially any drug, condition, or dietary change that causes a rapid rise in serum uric acid concentration through increased production or reduced secretion can precipitate or prolong an acute attack of gout. This includes starting or increasing urate-lowering therapy for the long-term management of gout or implementing other dietary changes for the management of gout, such as reducing alcohol consumption. Aspiration of an affected joint, bursa, or tophi is required to confirm the diagnosis of gout. Serum uric concentration should be measured in all patients with suspected gout [16].

## Conclusions

The prompt diagnosis, management, and prevention of hyperuricemia and gout are important to prevent further complications such as tophi formation, acute attacks, chronic kidney disease, and chronic destructive arthritis. Medical therapy along with lifestyle modification, avoidance of purine-rich foods, exercise, and halting the contributing drug and switching to a different class of medication is important to prevent recurrent gout attacks. Hyperuricemia is also associated with a higher incidence of NASH. The patient in our case required cessation of the culprit medication and dietary lifestyle modification to prevent further attacks.
